# What does complement do in Alzheimer’s disease? Old molecules with new insights

**DOI:** 10.1186/2047-9158-2-21

**Published:** 2013-10-12

**Authors:** Yong Shen, Libang Yang, Rena Li

**Affiliations:** 1 Center for Advanced Therapeutic Strategies for Brain Disorders, Roskamp Institute, 2040 Whitfield Avenue, Sarasota, USA; 2 Department of Pediatrics, School of Medicine, University of Minnesota, Minneapolis, MN 55455, USA; 3 Department of Neurology, College of Medicine, University of Florida, Gainesville, FL 32610, USA; 4 Center for Hormones Advanced Science and Education, Roskamp Institute, 2040 Whitfield Avenue, Sarasota, Florida, USA

## Abstract

Increasing evidence suggests that inflammatory and immune components in brain are important in Alzheimer’s disease (AD) and anti-inflammatory and immunotherapeutic approaches may be amenable to AD treatment. It is known that complement activation occurs in the brain of patients with AD, and contributes to a local inflammatory state development which is correlated with cognitive impairment. In addition to the complement’s critical role in the innate immune system recognizing and killing, or targeting for destruction, complement proteins can also interact with cell surface receptors to promote a local inflammatory response and contributes to the protection and healing of the host. On the other hand, complement activation also causes inflammation and cell damage as an essential immune function to eliminate cell debris and potentially toxic protein aggregates. It is the balance of these seemingly competing events that influences the ultimate state of neuronal function. Our mini review will be focusing on the unique molecular interactions happening in the AD development, the functional outcomes of those interactions, as well as the contribution of each element to AD.

## Introduction

The brain has been considered an immunologically privileged organ in part due to the presence of the blood–brain barrier (BBB), which prevents the entry of blood-born cells and other immune molecules from peripheral system into the central nervous system (CNS). However, multiple evidence indicates that this privilege is not absolute “sealed” from peripheral immune system. Studies demonstrated various immune and inflammatory mechanisms operating actively within the brain, particularly in response to disease or injury [1-10]. These findings have opened a new view of neuroimmunology and also brought great opportunities to develop novel agents that may prevent various neurodegenerative disorders as Alzheimer’s disease (AD), Parkinson’s disease (PD), multiple sclerosis (MS), AIDS dementia complex (ADC), amyotrophic lateral sclerosis (ALS) and stroke. This mini-review focuses only on the role of the complement system in AD.

Alzheimer’s disease (AD) is a chronic neurodegenerative disorder with characterization by extracellular senile plaques, intracellular neurofibrillary tangles and neuronal loss [11-15]. The major component of senile plaques is amyloid-β-peptide (Aβ), a group of 39–43 amino acid peptides derived from the amyloid precursor protein (APP) [11,12,16,17]. The fact that Aβ is detected in both normal and AD brains [16] indicates that Aβ alone may not be sufficient to cause AD. In recent years, the occurrence of inflammatory proteins in the AD brain has been widely reported [1,3-9,18-24]. One prominent feature of AD neuropathology is the association of activated proteins of the classical complement pathway with the lesions [1,5,25-28]. The full range of classical pathway complement proteins from C1q to C5b-9 (membrane attack complex, MAC), has been found highly localized with compacted or β-pleated Aβ deposits in neuritic plaques [4-7,26,28,29]. The complement cascades have been activated to the last step the MAC stage indicates that the regulatory mechanisms of the complement system have been unable to halt the complement activation process to avoid brain tissue. Some complement regulators have been found in association with the AD lesions [7,25,27,28,30,31]. This is a further proof of complement activation in the lesions but also an indication that the regulators have been able to control complement activation only to a limited extent.

Evidence for the involvement of inflammatory processes in the pathogenesis of AD have been documented for a long time [9,22,32-35]. From a therapeutic point of view, several direct studies on twins [32] and a recent “ibuprofen” clinical trial [34] have provided evidence that nonsteroidal anti-inflammatory drugs (NSAIDs) are one potential means of reducing inflammation in AD. Similar results were also obtained from a small double-blind, placebo-controlled trial using Indomethacin for AD prevention, a common NSAID [33,35]. Clinical trials of NSAIDs in AD patients have not been very fruitful lately. But in epidemiological studies that treatment with NSAIDs decreases the risk for developing AD [36]. NSAIDs are not very effective direct inhibitors of complement but they can inhibit the inflammatory consequences of complement activation, particularly those that are mediated by mast cells and other leukocytes [37].

## Roles of complement activation in alzheimer’s disease

Activation of any of three complement pathways (classical, lectin or alternative pathway) in the human body is very important in normal inflammatory responses to injury and in removing invading microbes. It is also a very important clean-up system in removing apoptotic cells, tissue debris and macromolecular aggregates. However, complement activation can also cause cell injury or death when activated inappropriately. Thus, although complement activation may not be the primary etiology of many diseases it can be responsible for many of the disease manifestations. Indeed, inappropriate complement activation has been recognized as an important pathogenetic factor in many cardiovascular, immune, renal and neurological diseases. In the nervous system, inflammation and neurodegeneration in AD are partially mediated by complement activation [1,5-7,26,38,39]. In comparison, in multiple sclerosis, which is a largely T-cell-mediated disease, complement seems to be important in causing demyelination and damage of oligodendrocytes [40,41].

### Complement cascade activated by pathological hallmarkers in Alzheimer’s brains

The complement cascade can be activated in the AD brain based on the evidence that Aβ-initiated, antibody-independent, complement activation in AD. The pioneer works were done by Rogers’ work and Tenner’s experiments by using Aβ binds C1q and activates the classical complement pathway [5,18,38,42,43]. This discovery provides preliminary a theory how inflammation arises and be sustained throughout the course of AD, since Aβ, possibly oligomer Aβ, is present from early stage to terminal stages of the disorder. Apparently, the classical pathway tries to remove the Aβ-protein deposits but fails in this task. Thus, continuing complement activation persists and causes inflammation. As discussed above, most studies determining inflammation characteristics for the AD brain have centered on the interactions of Aβ with complement proteins and other mediators of inflammation [44]. However, neurofibrillary tangles, the other classical pathological hallmark for AD, could play an equally important role in the neuropathology of AD. Our previous studies demonstrate that purified neurofibrillary tangles activate the complement system in plasma, resulting in a significant increase in the levels of soluble terminal complement complexes (SC5b-9) [8]. Like Aβ, the aggregated tau, a major component of neurofibrillary tangles, may also be a potent antibody-independent activator of the classical complement pathway [8,45].

The activated complement components have significant pro-inflammatory activities. For example, the complement C3 is cleaved into C3b and C3a. C3b remains bound to the complex at the surface of the trigger. C3b is a good opsonin. The small peptide, C3a diffuses away and acts as a chemotactic factor and an inflammatory paracrine. The generation of complement anaphylatoxins, notably C5a, activates leukocytes and induces the production of pro-inflammatory cytokines, which could be toxic or trophic depending on their target cells and receptor molecules [6,7,30,46-54]. The local production of complement proteins, from C1q to C9, has been found to be increased in the AD and other neurodegenerative disease brain [2,6,26,50,54-57]. Complement activation also results in the generation of other activation products, the opsonins, which label target cells for attack by phagocytes. The opsonins, C1q, C4b and C3b/iC3b, have been found in AD brain samples [18,20,27,30,57]. Furthermore, the formation of the complement membrane attack complex, the MAC, on the membranes of neuronal cells can result in their lysis [6,7,21,46]. Finally, MAC attack on neuronal and glial cells can also cause significant immunological activation of the cells with many unexpected consequences.

The significance of the complement activation in AD is not only the pathological changes in the terminal stage of AD but also reflects the early alternation in the disease course, i.e. mild cognitive impairment (MCI). For example, Loeffler et al. [58] have performed studies on iC3b, C9, Bielschowsky and Gallyas staining in in brains with 18 AD, 12 MCI with that from 17 aged normal controls and identified iC3b, C9, and Bielschowsky-stained plaque counts increased 2.5- to 3-fold in AD compared to MCI and control subjects. C9 staining was present on some diffuse plaques, as well as on neuritic plaques. Interestingly, Bielschowsky- complement-co-localized plaque counts were highly correlated. When the Bielschowsky plaque count was used as a predictor, its correlations with cognitive measures were statistically significant. This is a direct piece of pathological evidence linked with clinical assessment that the early-event complement molecule, iC3b, and late-event, final complement component protein, C9, appear in neocortical plaques in subjects across the cognitive spectrum; Moreover, C9 is observed in diffuse plaques. Due to high correlations between complement and Bielschowsky co-stained plaque counts, quantitative assessment of the extent to which complement activation may mediate the relationship between plaques and cognitive function. Recently Benoit et al. found that in mouse model, C1q pathways were up-regulated in vivo early in response to injury, induced a program of gene expression that promotes neuroprotection and thus might provide protection against Aβ in preclinical stages of AD and other neurodegenerative processes [59]. A potential clinical trial by using an inhibitor of late-stage complement activation, if there was any, in AD patients would be able to confirm the significance of this process in AD.

### Neurodegeneration mediated by complement activation and mechanisms

In vitro, Aβ binds C1q and activates the classical complement pathway [5,37,43,60]. Thereby Aβ can induce complement-mediated toxicity against neurons in culture [6,7,21,47]. This suggests that Aβ-induced complement activation may contribute to the neuropathogenesis in AD [4,25]. In APP+PS1 transgenic mice, Clq injections Increased fibrillar beta-amyloid [61]. MAC complexes have been detected in the AD brain, and their generation in vitro by Aβ stimulation leads to consequences that one would predict for a pathophysiologically relevant mechanism in AD [6,7].

Normally C5 convertases, generated by either the classical or the alternative pathway, initiate the activation of the terminal pathway of the complement system. This leads to the formation of the cytolytic MAC. The MAC is a macromolecular complex made up of complement components C5b, C6, C7 and C8 plus multiple C9 molecules. Once formed, the ring-like structure of the MAC constitutes a pore on the membrane of the target cells, permitting influx of Ca^2+^, Na^+^, small molecules and water. This can lead to subsequent osmotic cell lysis or to a metabolic “storm” within the cell. Neuroimmune regulatory proteins (NIReg) may control the adverse immune responses in health and diseases. NIRegs are found mainly on neurons, glia, endothelia and ependymal cells and include GPI-anchored molecules (CD24, CD90, complement regulators CD55 and CD59), molecules of the immunoglobulin superfamily (siglec CD22, Siglec 10, CD200, ICAM-5) and others (CD47, fractalkine, TAM receptor tyrosine kinase and complement C3a and factor H). These regulators modulate the innate immune response in the CNS and for instance critically control the level of phagocytosis and inflammation engaged by resident microglia and infiltrating immune cells [62].

Complement-containing human serum shows no significant toxicity against human neuronal cells, although it kills rat and mouse neurons [47]. As reported previously, the lack of susceptibility to human serum toxicity was apparently due to the presence of species-selective complement regulatory proteins such as complement inhibitors CD55, CD59 on the cell surface (a phenomenon referred to as homologous restriction). Since two complement inhibitors, CD55 and CD59, are glycosylphosphatidyl-inositol (GPI)-anchored cell membrane proteins, we examined the susceptibility of human neurotypic cells, SH-SY5Y, to human complement attack after treatment with phosphatidylinositol-specific phospholipase C (PIPLC). Indeed, human neuronal cells were susceptible to killing by human complement only after removal of GPI-anchored cell membrane proteins by PIPLC. In agreement with the LDH release measurements, the neurotoxic effect of human serum on PIPLC-pretreated cells was confirmed by morphological changes typical of injured neurons. One of the proteins removed by PIPLC is CD59, a membrane-bound glycoprotein capable of protecting against complement-mediated lysis by preventing the assembly of MAC [6,7,47,63-66]. As expected, PIPLC-pretreated SH-SY5Y neurotypic cells were also susceptible to human MAC-induced lysis and a significant increase in LDH release was observed after one day incubation with the equivalent of 4 hemolytic units of MAC [28,47]. These results suggested that neurons might be more vulnerable to complement-mediated Aβ toxicity when CD59 is dysfunctional or its levels on the cell surfaces are reduced.

To prevent MAC attack, CD59 forms a complex with C5b-8, thereby preventing further assembly of the poly-C9 MAC and its insertion into the cell membrane [63-65]. CD59 is widely distributed in human cells [6,7,27,47,63-65]. It has been found in many types of cells, including neurons, astrocytes, epithelial and endothelial cells [6,7,27,30,47,52,64,65]. Relative to other cell types, its expression by oligodendrocytes has been low. The mature CD59 protein contains 77 amino acids after removal of the signal sequence and the signal for the GPI-anchor. Its gene is located in the p14-p13 region on the short arm of chromosome 11 [66]. CD59 is attached to the cell membrane through a GPI-anchor, instead of having a transmembrane domain [67]. The site for the GPI-anchor attachment of CD59 is the most carboxy-terminal amino acid Asn-77 [63]. As discussed above, MAC inhibition by CD59 occurs if the MAC components are from the same species as the target cell [47,63]. Conversely, when the complement source and the target cells are from different species, CD59 may not always inhibit the MAC. Because of this species-selectivity, complement-mediated lysis of cells can occur, if the cells and complement are from different species. We observed CD59 expression in neuronal cells by detecting specific CD59 DNA by an oligonucleotide probe in PCR-Southern blot hybridization. The expression of the CD59 protein in neurotypic cells was confirmed by immunofluorescence staining. The expression became significantly reduced upon PIPLC treatment [30,47]. Gasque’s and Morgan’s groups [68,69] observed that human fetal neurons expressed some CD59, but were expressed much higher levels in astrocytes [48,68,70]. Thus, human fetal neurons appear to be more vulnerable to complement activation than astrocytes. Decreased levels or absent CD59 may permit MAC formation and lead to nonspecific complement lysis. This is the case in paroxysmal nocturnal hemoglobinuria (PNH), where a proportion of bone marrow-derived cells lack GPI-anchored CD59 and CD55. Our recent studies suggest that similar conditions are present in the AD brain. As a consequence, increased MAC formation, deposition and cell lysis as a result of a deficient CD59 regulatory activity could play a pathophysiologic role in AD neurodegeneration [6,7].

Because of the cell stimulating and destructive capabilities of the MAC, a rigorous analysis of reasons for its formation in the AD brain is extremely important. Also, the extent of expression and properties of CD59 in the AD brain must be defined, since a deficiency in CD59 could render even moderate MAC formation, a potent neurodegenerative mechanism. Although a cellular source for MAC components has not been conclusively defined, the identification, both of proteins and mRNAs, of MAC components in the AD brain suggests that a proportion of them could be endogenously produced [6,7,20,26,51]. Since some of the complement components (e.g., C1q) are relatively large in size and charged [60], their passage from blood through an intact BBB could be restricted. *In vitro*, astrocytoma cell lines and primary astrocytes, as well as microglia, have been shown to produce complement proteins, suggesting that glial cells may be an endogenous source within the brain [6,7,70,71]. This is especially true for microglial cells, since they are closely related to macrophages, a cell type known to manufacture complement components [72]. Perhaps surprisingly, recent evidence [51,70], including that from our laboratory [6,7,26], suggests that neurons and certain neuroblastoma lines may produce virtually the full range of classical pathway components. To what end they might do so, and under what conditions, is still unclear.

Virtually all cells in the body can become targets for complement attack. So they must have developed a relatively wide range of defenses, including CD59 expression. From this concept, we have discovered phenomena so called “homologous restriction” between human species and other rodent species [26], which protect our human being protect ourselves against self-complement attack [45]. Therefore, endogenous CD59 production by both neurons and glia in the brain would be expected because of “homologous restriction” we just discussed above. It is true that complement regulatory proteins can be found in the brain parenchyma and are increased, especially under acute inflammatory conditions, i.e. meningitis. However, in the condition for chronic inflammatory case, i.e. AD, the expression levels of complement regulatory proteins may be at a low level. We previously discovered that CD59 is deficient in the AD brains [7] and believe that this possibly dues to either inflammatory molecules down-regulate complement regulatory proteins or some endogenous enzymatic-like molecules cleave of the GPI-anchored proteins, including CD59 [7], which allow complement cascades are activated, leading to sustained activation of glial cells or neuron death or both.

### Potential therapeutic targets to complement activation in Alzheimer’s disease

Based upon these immunological findings, complement inhibitors could possibly provide an alternative therapeutic means as neuroprotective agents or as anti-neuroinflammatory agents in treating AD or other CNS disorders, where complement activation is involved. They can act at different levels of complement activation and have distinct properties. In the following, a couple of examples are provided.

#### Inhibition of the serine proteases of the complement system

The complement system is a proteolytic cascade, where serine proteases activate each other by limited proteolysis in a strictly ordered manner. Serine proteases are essential in both the initiation and the amplification of the cascade. Since uncontrolled complement activation contributes to the development of serious disease conditions, inhibition of the complement serine proteases could be an attractive therapeutic approach. Major types of serine protease inhibitors include (a) macroglobulins, (b) serpins and (c) canonical inhibitors. Macroglobulins and serpins are large proteins which undergo major conformational change during the inhibition process and form irreversible complex with the target proteases. The canonical inhibitors are small proteins which occupy the active site and form very tight but thermodynamically reversible complex with the target proteases. C1-inhibitor (C1INH) is a good example. C1INH a natural complement protease inhibitor, which is approved for clinical use in hereditary angioedema (HAE) [73]. Sequence comparisons and functional analyses have indicated that C1INH α_1_-antitrypsin, α_2_-antiplasmin, antithrombin III and plasminogen activator inhibitor types I and III belong to the superfamily of serine protease inhibitors or serpins [74]. C1INH is a plasma glycoprotein of 105 kD with gene location on chromosome 11 [75]. C1INH can inhibit components C1r and C1s of the classical pathway of complement activation through the formation of an inhibitor-protease complex [76]. The complex is formed between the reactive center of the C1INH amino-terminus and the active site of the protease. In the central nervous system, C1INH has been identified in neurons. Like in other serpins, the reactive region of serpins mimics the substrate of the protease. One hypothesis proposes [31,52] that inactivation of the C1INH may play a role in local inflammatory reactions and pathological circumstances. The level of C1INH is decreased in type I (HAE), a disorder inherited in an autosomal dominant fashion. In patients with type II HAE, mutated dysfunctional C1INHs are found [52]. In the AD brain, both C1INH mRNA and protein have been identified [19,31]. C1INH has not only been found in neurons but also in microglial cells and astrocytes [31]. Interestingly, these studies have demonstrated that C1INH is present in inactive form in activated microglia and astrocytes. Moreover, C1INH has also been detected in abnormal neuronal processes, such as dystrophic neurites in the AD brain [31]. Eikelenboom [39] and Veerhuis [19] found that interferon-gamma stimulated C1INH protein secretion in the AD brain but IL-1β, IL-6 and TNF-α could only stimulate C1INH synthesis at the mRNA, but not at the protein level [1,39]. Thus, a block in C1INH synthesis could occur at the level of translation. Moreover, *in situ* hybridization studies demonstrated that C1INH mRNA was primarily expressed in neurons, indicating that neurons may be an important source for complement regulatory proteins *in vivo*. Because of its biological properties C1INH may be used as a possible therapeutic agent. C1INH preparations have been used e.g. in the treatment and prevention of HAE attacks, and to suppress inflammation in the ischemia-reperfusion syndrome that is associated with myocardial infarction [77].

#### Complement receptor type 1 (CR1, C3b/C4bC receptor or CD35)

Complement receptor type 1 (CR1) can be detected on blood cell surfaces by immunofluorescence imaging techniques. For example, counting CR1 numbers in granulocytes by FACS, is used for monitoring the extent of systemic neutrophil and monocyte activation [45]. A soluble form of CR1 (sCR1) can be detected in serum at the ng/ml level, a concentration that already can inhibit pathological complement activation. Because of its potency, recombinant soluble CR1 has been considered as a complement-inhibiting drug [48,70]. In the central nervous system, administration of sCR1 in rats resulted in a 40% decrease in brain neutrophil accumulation in the traumatized hemisphere as compared to normal controls [52,78]. sCR1 was first studied in myocardial infarction [79] and it has been suggested as a therapy to prevent rejection of xenotransplants and in various inflammatory diseases. Thus, sCR1 is a feasible and potent complement inhibitor, of which experience on use *in vivo* is available.

Recently, genome-wide association studies (GWAS) identified that the CR1-S isoform has been associated with AD and is considered a novel AD gene [80]. The analyses of brain samples demonstrated that the CR1-S isoform protein expression is at lower protein levels than CR1-F (p < 0.0001) hence likely associated with increased complement activation [81]. Interestingly, the pathological results demonstrate the different expression patterns of CR1 in neurons between the F/F and F/S genotypes. Moreover, double-labeling studies supported such differential distributions of CR1 in endoplasmic reticulum intermediate compartment compared to lysosomes in neurons. These findings suggest that the CR1-S and CR1-F isoforms process differentially in different ways in neurons and provide a novel prospect for the investigation of CR1-related mechanisms for AD.

#### Proteoglycans as inhibitors of C1q binding

Proteoglycans are heavily glycosylated proteins. They are a type of molecule found in connective tissue and considered as potential therapeutic molecules for many diseases. C1q combines with the enzymes C1r and C1s to form C1, the first component of the classical complement pathway [82]. C1q can bind to immunoglobulins and to non-immunoglobulin classical pathway activators such as the Aβ peptide [2,5,18,39,43,60,83]. C1q also binds to apoptotic cells and substances released from injured cells, like mitochondria, cytoskeletal filaments and chromatin. C1q binding initiates activation of the classical complement cascade, which ultimately leads to MAC formation and target cell lysis [6,21,47]. C1q deposits are often found in injured tissues. Thus, it would appear logical to design and develop drugs to block C1q binding so that the complement cascade is inhibited at an early stage [84,85]. Chondroitin sulfate proteoglycans (CSPG) have recently been found in human B cell line supernatants [86]. The secreted CSPGs bound strongly to C1q but cellular CSPGs did not. The binding of CSPG to solid-phase-bound C1q could inhibit formation of the C1 complex with C1r and C1s. Thus, CSPG could offer a tool for inhibiting C1 activation, but so far, all experiments have been done only *in vitro*. CSPG has a broad spectrum of activities. Another drawback is that inhibition of C1q binding could compromise the normal cleaning function of the classical pathway. It is well known that C1q deficiency in man and experimental gene knock-out animals leads to a severe form of systemic lupus-like disease [87].

#### Inhibitors of C3 convertase

FUT-175 (nafamstat) is a potent synthetic serine protease inhibitor. It has potent anti-complement activity *in vitro.* It can inhibit the activity of C1r and C1s proteases but has no inhibitory effect on the C2a protease activity [2]. Clinically, the administration of FUT-175 intravenously to patients with complement activation associated glomerulonephritis resulted in a significant decrease in urinary protein excretion and in an increase in the levels of serum complement proteins C3 and C4 [88]. Syringin (TC-4) and cordiol (TC-7), derived from an Indian plant, also have anticomplementary and immunomodulatory activities. Recently, it was discovered that these two compounds inhibit the C3 convertase of the classical complement pathway [89,90]. Recently Holmquist et al. discovered Sushi domain-containing protein 4 (SUSD4) is a novel complement inhibitor and it inhibited the formation of the classical C3 convertase by 90% [91].

Other putative complement inhibitors such as fucans, naturally sulfated polysaccharides, have been isolated from brown seaweed [92,93]. Fucans inhibit the classical pathway by interfering with C1 activation or by inhibiting C3 cleavage [94] by the classical pathway C3 convertase. They may also inhibit the alternative pathway C3 convertase by suppressing factor B binding to C3b and destabilizing properdin function [94]. However, fucans have no effect on the formation of the MAC [94].

Cerebral amyloid angiopathy (CAA) has similar AD pathologies associated with Aβ accumulation and inflammation in the brain. Zabel et al. [95] has examined human post-mortem brains with concomitant CAA and AD with purely parenchymal pathology and for differential expression of microglia-associated Aβ ligands thought to mediate Aβ clearance and the association of these receptors with complement activation. They found that C3b and MAC were significantly increased in CAA compared to AD-only and controls and immunoprecipitation (IP) showed significantly increased CD11b/C3b complexes (in microglia) with Aβ in AD/CAA subjects. Immunohistochemical studies with confocal microscopy reveal these interactions. MAC was remarkably associated with CAA-affected blood vessels compared to AD-only and control vessels. These findings suggest an Aβ clearance mechanism via microglial CD11b that delivers Aβ and C3b to blood vessels in CAA (maybe AD as well), which leads to Aβ accumulation and propagation of complement to the cytolytic MAC, possibly leading to vascular fragility [95].

Microglia is a microphage of the brain, and abnormal activation of microglia in cascades result in neuronal loss and cognitive decline in AD [72]. As described before, Recent GWAS have indicated a number of risk factors, CR1, for the development of late-onset AD, which may implicate microglial responses in AD during the course of complement activation in the brain. Changing complement receptor expression may result in disorder of the complement activation cascade, no matter over-activated different complement pathways or imbalances between complement factor production and complement cascade inhibitors, which may contribute to the involvement of complement in AD. Moreover, abnormal complement signaling may reduce the ability of microglia to phagocytose apoptotic cells and clear Aβ peptides, modulate the expression by microglia of complement components and receptors, promote complement factor production by plaque-associated cytokines derived from activated microglia and astrocytes, and disrupt complement inhibitor production.

#### C5 activation blockers or C5a receptors

The C5 activation product, C5a, is a 4-helix bundle glycoprotein containing 74 amino acids. It is a strong chemotactic agent for neutrophils, macrophages and microglia and activates mediator release from many cell types, notably from mast cells. C5a exerts its primary physiological and pathological effects by binding to its specific G-protein coupled C5a receptor (C5aR; CD88). This leads to triggering of signal transduction and powerful inflammatory responses [96-98]. The presence of C5a receptors on neurons and glia in the brain raises the possibility that they might respond to locally generated C5a [7,26,68,70,71,99]. Gasque et al. found that the C5a receptor is expressed in the human brain and particularly in astrocytes [48]. Because C5a is an important mediator of inflammation, C5a receptor antagonists could have therapeutic potential. Recently, a series of high-affinity, basic benzodiazepine ligands for the C5a receptor have been developed [100]. Because these ligands are more basic (pKa = 9.48) than the previous ligands, their aqueous solubility has been significantly improved. The IC_50_ values of these compounds for C5aR are < 2.5 nM. However, the inhibitory effects of benzodiazepines on C5a receptor activity have not been extensively reported, neither *in vitro* nor *in vivo*. To specifically inhibit C5aR, a small peptide derived from the C5a hexapeptide C terminus has been recently reported [101]. Analyses of the antagonist’s tertiary structure and the effects of point mutations demonstrate a positively charged contact surface composed of Arg 75, Arg 46, Lys 49 and His 15 residues. The importance of this surface in providing antagonist properties implies a single binding site in the C5a receptor protein [102]. It would be important to examine whether these compounds have any effects in the central nervous system.

One of the most promising specific complement inhibitors for clinical use is the humanized anti-C5 antibody, pexelizumab, produced by the Alexion corp [103]. This antibody blocks the cleavage of C5, thereby inhibiting both C5a generation and MAC assembly, which is initiated upon C5b formation. Importantly, the earlier complement cascade up to the C3 level is left untouched. This means that early classical pathway-mediated solubilization of protein aggregates, possibly including also those of Aβ, and opsonization for phagocytosis are left intact. Theoretically, this antibody would appear as ideal for suppressing C5a- and MAC-mediated inflammation, although it may not help in the solubilization or removal of Aβ-aggregates by the earlier parts of the complement cascade.

Since AD is associated with neuroinflammation, activation of astrocytes and microglia, and evidence of activation of the complement system, localized with both fibrillar Ab (fAb) plaques and tangles. Using the compound PMX205 to inhibit the major complement receptor for C5a (CD88) leads to less pathology in mouse models of AD. While thioflavine plaque load and glial recruitment is significantly reduced after treatment with PMX205, C1q remains co-localized with fAbeta plaques and C3 is still expressed by the recruited astrocytes. Thus, with PMX205, potentially beneficial activities of these early complement components may remain intact, while detrimental activities resulting from C5a-CD88 interaction are inhibited [104]. This further supports the targeted inhibition of specific complement mediated activities as an approach for AD therapy.

#### Neuroimmune regulatory proteins

NIReg contributes to the adverse immune responses in health and diseases. NIRegs are found mainly on neurons, glia, endothelia and ependymal cells. They include sialic acids, GPI-anchored molecules (CD24, CD90, complement regulators CD55 and CD59), molecules of the immunoglobulin superfamily (siglec CD22, Siglec 10, CD200, ICAM-5) and others (CD47, fractalkine, TAM receptor tyrosine kinase and complement C3a and factor H). These regulators contribute to control the innate immune response in the CNS. Some of NIRegs could be potential therapeutical molecules [62,105-108]. Griffith et al. found that accumulation of human factor H in the brain parenchyma protected neurons from complement opsonization, axonal injury, and leukocyte infiltration [109]. Axonal damage secondary to inflammation is found in the animal model of experimental autoimmune encephalomyelitis (EAE). Wld(s) mice have a triplication of the fusion gene Ube4b/Nmnat and a phenotype of axon protection. Wld(s) mice develop an attenuated disease course of EAE, with decreased demyelination, reduced axonal pathology, and decreased CNS macrophage and microglial accumulation. The attenuated disease in Wld(s) mice was associated with higher expression of the nonsignaling CD200 molecule on neurons in the CNS compared with control mice. In vitro, Wld(s) neuronal cultures were protected from microglial-induced neurotoxicity compared with control cultures, but protection was blocked by anti-CD200 antibody. CD200 interacts with its signaling receptor CD200R. Then CD200-CD200R pathway plays a critical role in attenuating EAE and reducing inflammation-mediated damage in the CNS. Strategies that up-regulate the expression of CD200 in the CNS or molecules that ligate the CD200R may be relevant as neuroprotective strategies in multiple sclerosis [105].

Decay-accelerating factor (DAF, CD55) inhibits complement activation by suppressing the function of C3/C5 convertases, thereby limiting local generation or deposition of C3a/C5a and MAC production. When compared to controls, hypoxic cells had fewer dendritic spines, reduced plateau depolarization accompanied by increased apoptotic activity and accumulation of MAC, as well as up-regulation of C3, C3a and C3aR, enhancement of C3a-C3aR engagement, and elevated caspase and Src activity. Treatment of hypoxic cells with 200 ng/ml of recombinant human DAF resulted in attenuation of neuronal apoptosis and exerted significant protection against neuronal dendritic spine loss and plateau depolarization reduction. Furthermore, treatment with DAF resulted in decreased accumulation of C3a, MAC, C3a-C3aR interaction, caspase-9, activated caspase-3, and pTyr416-Src (activated Src) tyrosine kinase. DAF was found to reduce neuronal cell death and apoptosis in NaCN induced hypoxia [107].

CD59 expression is regulated by the neural-restrictive silencer factor (REST). A designed novel REST-derived peptide (REST5) containing the nuclear localization domain of the wild-type protein was used to observe this regulation. REST5 increased the expression of CD59 in neurons by fivefold and protected them from complement-mediated lysis spontaneously triggered by neurons [108].

## Complement proteins as potential biomarkers and vaccination of AD

### Complement proteins may use potential biomarkers for AD

As previous described, the complement cascade is an essential element of the innate immune response. In the brain complement proteins are integral components of plaques (i.e. C1q binds Aβ) or tangles (aggregated tau binds C1q). Complement activation can occur at the very early stage of the disease. Therefore, certain complement components for complement activation act as potential biomarker for AD during the disease process [110].

Wang et al. [111] recently examined complement 3 and factor H (alternative complement pathway complement factor) in human cerebrospinal fluid in AD, compared to other neurological controls such as PD and multiple-system atrophy. Interestingly, they found that both C3 and FH correlated with the severity of cognitive impairment in AD.

A study was conducted recently with four single nucleotide polymorphisms (SNPs) in complement genes and cerebrospinal fluid (CSF) biomarkers for AD in 452 neurochemically or neuropathologically verified AD cases and 678 cognitively normal controls. None of the SNPs associated with risk of AD but there were potential associations of rs9332739 in the C2 gene and rs4151667 in the complement factor B gene with CSF tau levels (p = 0.023) and Mini-Mental State Examination scores (p = 0.012), both of which may be considered markers of disease intensity/severity [112].

Because there is a deficiency of CD59 GPI anchored proteins in AD brains [7] due to the cleavage of these proteins from brain cells, it is possible that the CD59 proteins flow into the CSF and plasma when the blood–brain-barrier BBB is damaged in AD patients. We examined CD59 levels in CSF from postmortem patients and found an increase compared to ND controls (Figure X). Conversely, complement C9 component is the final component to be added into the C5b-8 subcomplex of MAC. We previously discovered that C9 levels were significantly elevated in the same regions where the CD59 protein is deficient in AD brains [7]. If the final complement component C9 is heavily deposited in AD brains, the AD brain acts like a toxin sink to trap many toxins, including C9, resulting in low levels of C9 in the CSF. Indeed, we detected the decrease of C9 protein level from postmortem patients with AD (Figure XX). Identifying an accurate biomarker that has sufficient predictive, diagnostic and prognostic value would provide a significant opportunity to develop and test for effective novel therapies in the treatment of AD [111,113].

### Vaccination as an alternative treatment of Alzheimer’s disease

Amyloid beta protein plays a pivotal role in AD onset and progression and secondary consequences of Aβ generation and deposition, including tau hyperphosphorylation and neurofibrillary tangle formation, oxidation, inflammation, and excitotoxicity, contribute to the disease process. Interventions in these processes with agents that reduce amyloid production, limit aggregation, or increase removal or vaccination and immunization might block the cascade of events comprising AD pathogenesis [114]. In the past few years, studies on experimental vaccines have been conducted [69,99,115-117]. A number of passive immunizations with anti-Aβ42 antibodies are in different phases of clinical trials. One active immunization approach, AN-1792 (consisting of preaggregate Aβ and an immune adjuvant, QS-21), was stopped after the development of autoimmune encephalitis in 6% of patients and a second one, CAD106, in which a small Aβ epitope is used, is currently in safety and tolerability studies. Besides active immunizations with proteins or peptides, active immunizations using DNA which codes for the protein against which the immune response will be directed, so called genetic immunizations, provide additional safety as the immune response in DNA immunizations differs quantitatively and qualitatively from the response elicited by peptide immunizations [117-121]. Anti-Aβ monoclonal antibodies (bapineuzumab and solanezumab) are now being developed. The clinical results of the initial studies with bapineuzumab were equivocal in terms of cognitive benefit. Solanezumab, a humanized anti-Aβ monoclonal antibody directed against the midregion of the Aβ peptide, was shown to neutralize soluble Aβ species. Phase II studies showed a good safety profile of solanezumab, while studies on cerebrospinal and plasma biomarkers documented good signals of pharmacodynamic activity. The results of the large, ongoing Phase III trials with bapineuzumab and solanezumab will tell us if monoclonal anti-Aβ antibodies may slow down the rate of deterioration of AD [118-123]. As we described above, one theory of the cause of AD is the inflammation hypothesis, whereby Aβ deposits in the brain induce an inflammatory response that activate microglia to produce toxins and destroy surrounding neurons, which results in a cognitive decline. There are three possible working hypotheses for this immunization under debate. For the first hypothesis, antibody binds to Aβ deposits and activates the complement system, which in turn triggers receptors on microglial cells to begin phagocytosis and remove debris or internalize Aβ [118-120]. Secondly, vaccines or antibodies dissolve the Aβ containing plaques directly, which would release monomeric Aβ, causing activation of microglial cells through some scavenger receptors on the surface of microglia. Thirdly, antibodies act as an “Aβ sink” in the peripheral system to enhance clearance of Aβ [124-126]. These possibilities have been comprehensively reviewed from different aspects in recent reviews [118,121-123,126-128]. The main message of this mini-review is that complement can have dual effects in Alzheimer’s disease. In the negative aspect complement activation by Aβ or tau causes neurodegeneration, whereas in the positive aspect complement is important for the tissue clearance functions. Since these functions are partially mediated by different parts of the complement cascade, any therapeutic interventive approaches should be appropriately focused and thoroughly tested before use.

## Competing interests

All authors in this paper declare no competing financial interests.

## Authors’ contributions

YS and LY have written the manuscript draft, RL’s idea to such review article and overview the review article writing process. All authors read and approved the final manuscript.

## References

[B1] Eikelenboom P Stam FC Immunoglobulins and complement factors in senile plaques Acta Neuropath Berl 1982 57 239 242 10.1007/BF00685397 6812382

[B2] Haga S Ikeda K Sato M Ishii T Synthetic Alzheimer amyloid beta/A4 peptides enhance production of complement C3 component by cultured microglial cells Brain Res 1993 601 88 94 8431789 10.1016/0006-8993(93)91698-r

[B3] Griffin WS Sheng JG Roberts GW Mrak RE Interleukin-1 expression in different plaque types in Alzheimer’s disease: significance in plaque evolution J Neuropath Exp Neurol 1995 54 276 281 7876895 10.1097/00005072-199503000-00014

[B4] McGeer PL Akiyama H Itagaki S McGeer EG Activation of the classical complement pathway in brain tissue of Alzheimer patients Neurosci Lett 1989 107 341 346 2559373 10.1016/0304-3940(89)90843-4

[B5] Rogers J Cooper N Webster S Schultz J McGeer P Styren SD Civin WH Brachova L Bradt B Ward P Lieberburg I Complement activation by β-amyloid in Alzheimer’s disease Proc Natl Acad Sci USA 1992 89 10016 10020 1438191 10.1073/pnas.89.21.10016 PMC50268

[B6] Shen Y Sullivan T Meri S Shiosaki K Lin CW Induced expression of neuronal membrane attack complex (MAC) and cell death by beta-amyloid peptide Brain Res 1998 796 187 197 9689469 10.1016/s0006-8993(98)00346-1

[B7] Yang LB Li R Meri S Rogers J Shen Y Deficiency of complement defense protein, CD59, may contribute to neurodegeneration of Alzheimer’s brains J Neurosci 2000 20 1600 1618 10.1523/JNEUROSCI.20-20-07505.2000 PMC6772855 11027207

[B8] Shen Y Lue L Yang L Roher A Kuo Y Stromeyer R Goux WJ Lee V Johnson GV Webster SD Cooper NR Bradt B Rogers J Complement activation by neurofibrillary tangles in Alzheimer’s disease Neuroscience letter 2001 305 165 168 10.1016/s0304-3940(01)01842-0 11403931

[B9] Broussard GJ Mytar J Li RC Klapstein GJ The role of inflammatory processes in Alzheimer’s disease Inflammopharmacology 2012 20 109 26 22535513 10.1007/s10787-012-0130-z

[B10] Luo XG Chen SD The changing phenotype of microglia from homeostasis to disease Transl Neurodegeneration 2012 1 9 10.1186/2047-9158-1-9 PMC3514090 23210447

[B11] Price DL Sisodia SS Toxicity of synthetic A beta peptide and modeling of Alzheimer’s disease Ann Neurosci 1998 13 623 625 10.1016/0197-4580(92)90069-a 1461354

[B12] Selkoe DJ Yamazaki T Citron M Podlisny MB Koo EH Teplow DB Haass C The role of APP processing and trafficking pathways in the formation of amyloid beta-protein Ann NY Acad Sci 1996 777 57 64 8624127 10.1111/j.1749-6632.1996.tb34401.x

[B13] Sisodia SS Gallagher M A role for the beta-amyloid precursor protein in memory? Proc Natl Acad Sci U S A 1998 95 12074 12076 9770440 10.1073/pnas.95.21.12074 PMC33903

[B14] Di Carlo M Giacomazza D San Biagio PL Alzheimer’s disease: biological aspects, therapeutic perspectives and diagnostic tools J Phys: Condens Matter 2012 24 244102 22595372 10.1088/0953-8984/24/24/244102

[B15] Medeiros R Chabrier MA LaFerla FM Elucidating the triggers, progression, and effects of Alzheimer’s disease J Alzheimers Dis 2013 33 Suppl 1 S195 210 22635105 10.3233/JAD-2012-129009

[B16] Ashall F Goate AM Role of the beta-amyloid precursor protein in Alzheimer’s disease Trends in Biochem Sci 1994 19 42 46 8140621 10.1016/0968-0004(94)90173-2

[B17] Honjo K Black SE Verhoeff NP Alzheimer’s disease, cerebrovascular disease, and the β- amyloid cascade Can J Neurol Sci 2012 39 712 28 23227576 10.1017/s0317167100015547

[B18] Afagh A Cummings B Cribbs D Cotman CW Tenner AJ Localization and cell association of C1q in Alzheimer’s disease brain Exp Neurol 1996 138 22 32 8593893 10.1006/exnr.1996.0043

[B19] Veerhuis R Janssen I Hoozemans JJ De Groot CJ Hack CE Eikelenboom P Complement C1INH expression in Alzheimer’s disease Acta Neuropathol (Berl) 1998 96 287 296 9754962 10.1007/s004010050896

[B20] Walker DG McGeer PL Complement gene expression in human brain: comparison between normal and Alzheimer disease cases Mol Brain Res 1992 14 109 116 1323007 10.1016/0169-328x(92)90017-6

[B21] Webster S Lue LF Brachova L Tenner AJ McGeer PL Terai K Walker DG Bradt B Cooper NR Rogers J Molecular and cellular characterization of the membrane attack complex, C5b-9, in Alzheimer’s disease Neurobiol Aging 1997 18 415 421 9330973 10.1016/s0197-4580(97)00042-0

[B22] Azizi G Mirshafiey A The potential role of proinflammatory and antiinflammatory cytokines in Alzheimer disease pathogenesis Immunopharmacol Immunotoxicol 2012 34 881 95 22970774 10.3109/08923973.2012.705292

[B23] Michaud M Balardy L Moulis G Gaudin C Peyrot C Vellas B Cesari M Nourhashemi F Proinflammatory cytokines, aging, and age-related diseases J Am Med Dir Assoc 2013 Doi: 10.1016/j 10.1016/j.jamda.2013.05.009 23792036

[B24] Quintanilla RA Orellana JA von Bernhardi R Understanding risk factors for Alzheimer’s disease: interplay of neuroinflammation, connexin-based communication and oxidative stress Arch Med Res 2012 43 632 44 23142264 10.1016/j.arcmed.2012.10.016

[B25] Eikelenboom P van Exel E Veerhuis R Rozemuller AJ van Gool WA Hoozemans JJ Innate immunity and the etiology of late-onset Alzheimer’s disease Neurodegener Dis 2012 10 271 3 22261470 10.1159/000334287

[B26] Shen Y Li R McGeer E McGeer P Neuronal expression of mRNAs for complement proteins of the classical pathway in Alzheimer’s brain Brain Res 1997 769 391 395 9374212 10.1016/s0006-8993(97)00850-0

[B27] McGeer PL Walker DG Akiyama H Kawamata AL Guan AL Parker CJ Okada N McGeer EG Detection of the membrane inhibitor of reactive lysis (CD59) in diseased neurons of Alzheimer’s brain Brain Res 1991 544 315 319 1710165 10.1016/0006-8993(91)90071-3

[B28] Timmer NM Kuiperij HB de Waal RM Verbeek MM **Do amyloid β-associated factors co-deposit with Aβ in mouse models for Alzheimer’s disease**? J Alzheimers Dis 2010 22 345 55 20847441 10.3233/JAD-2010-100711

[B29] O’Barr SA Caguioa J Gruol D Perkins G Ember JA Hugli T Cooper NR Neuronal expression of a functional receptor for the C5a complement activation fragment J Immunol 2001 166 4154 4162 11238666 10.4049/jimmunol.166.6.4154

[B30] Singhrao SK Neal JW Rushmere NK Morgan BP Gasque P Spontaenous classical pathway activation and deficiency of membrane regulators render human neurons suseptible to complement lysis Am J Pathol 2000 157 905 918 10980130 10.1016/S0002-9440(10)64604-4 PMC1885712

[B31] Walker DG Yasuhara O Patston PA McGeer EG McGeer PL Complement C1INH is produced by brain tissue and is cleaved in Alzheimer disease Brain Res 1995 675 75 82 7796155 10.1016/0006-8993(95)00041-n

[B32] Breitner JC Gau BA Welsh KA Plassman BL McDonald WM Helms MJ Anthony JC Inverse association of anti-inflammatory treatments and Alzheimer’s disease: initial results of a co-twin control study Neurology 1994 44 227 232 8309563 10.1212/wnl.44.2.227

[B33] McGeer PL Schuler M McGeer EG Arthritis and anti-inflammatory agents as possible protective factors for Alzheimer’s disease: a review of 17 epidemiologic studies Neurology 1996 47 425 432 8757015 10.1212/wnl.47.2.425

[B34] Rich JB Rasmusson DX Folstein MF Carson KA Kawas C Brandt J Nonsteroidal anti-inflammatory drugs in Alzheimer’s disease Neurology 1995 45 51 55 7824134 10.1212/wnl.45.1.51

[B35] Rogers J Kirby L Hempelman S Berry DL McGeer PL Kaszniak AW Zalinski J Cofield M Mansukhani L Wilson P Kogan F Clinical trial of indomethacin in Alzheimer’s disease Neurology 1993 43 1609 1611 8351023 10.1212/wnl.43.8.1609

[B36] Rubio-Perez JM Morillas-Ruiz JM A review: inflammatory process in Alzheimer’s disease, role of cytokines Sci World J 2012 2012 756357 10.1100/2012/756357 PMC3330269 22566778

[B37] Heneka MT Kummer MP Weggen S Bulic B Multhaup G Münter L Hüll M Pflanzner T Pietrzik CU Molecular mechanisms and therapeutic application of NSAIDs and derived compounds in Alzheimer’s disease Curr Alzheimer Res 2011 8 115 31 21345168 10.2174/156720511795256099

[B38] Webster SD Galvan MD Ferran E Garzon-Rodriguez W Glabe CG Tenner AJ Antibody-mediated phagocytosis of the amyloid beta-peptide in microglia is differentially modulated by C1q J Immunol 2001 166 7496 7503 11390503 10.4049/jimmunol.166.12.7496

[B39] Eikelenboom P Zhan S van Gool W Allsop D Inflammatory mechanisms in Alzheimer’s disease Trends Pharmacol Sci 1994 15 447 450 7886816 10.1016/0165-6147(94)90057-4

[B40] Rollins SA Zhao J Ninomiya H Sims PJ Inhibition of homologous complement by CD59 is mediated by a species-selective recognition conferred through binding to C8 within C5b-8 or C9 within C5b-9 J Immunol 1991 146 2345 51 1706395

[B41] Storch MK Piddlesden S Haltia M Iivanainen M Morgan P Lassmann H Multiple sclerosis: in situ evidence for antibody- and complement-mediated demyelination Ann Neurol 1998 43 465 471 9546327 10.1002/ana.410430409

[B42] Jiang H Burdick D Glabe CG Cotman CW Tenner AJ Beta-Amyloid activates complement by binding to a specific region of the collagen-like domain of the C1q A chain J Immunol 1994 152 5050 5059 8176223

[B43] Velazquez P Cribbs DH Poulos TL Tenner AJ Aspartate residue 7 in amyloid beta-protein is critical for classical complement pathway activation: implications for Alzheimer’s disease pathogenesis Nat Med 1997 3 77 79 8986745 10.1038/nm0197-77

[B44] Ishii T Haga S Kametani F Pouplard-Barthelaix A, Emile J, Christen Y **Presence of immunoglobulins and complements in the amyloid plaques in the brain of patients with Alzheimer’s disease** Immunology and Alzheimer’s disease 1988 Berlin: Springer-Verlag 17 29

[B45] Shen Y Meri S Yin and Yang: complement activation and regulation in Alzheimer’s disease Prog Neurobiol 2003 70 463 72 14568360 10.1016/j.pneurobio.2003.08.001

[B46] Duan Y Dong S Gu F Hu Y Zhao Z Advances in the pathogenesis of Alzheimer’s disease: focusing on tau-mediated neurodegeneration Transl Neurodegeneration 2012 1 24 10.1186/2047-9158-1-24 PMC3598890 23241453

[B47] Shen Y Halperin JA Lee CM Complement-mediated neurotoxicity is regulated by homologous restriction Brain Res 1995 671 282 292 7743216 10.1016/0006-8993(94)01264-i

[B48] Gasque P Chan P Fontaine M Ischenko A Lamacz M Gotze O Morgan BP Identification and characterization of the complement C5a anaphylatoxin receptor on human astrocytes J Immunol 1995 155 4882 4889 7594492

[B49] Kirschfink M Targeting complement in therapy Immunol Rev 2001 180 177 189 11414360 10.1034/j.1600-065x.2001.1800116.x

[B50] Mulligan MS Yeh CG Rudolph AR Ward PA Protective effects of soluble CR1 in complement and neutrophil-mediated tissue injury J Immunol 1992 148 479 485 1311349

[B51] Van Beek J Bernaudin M Petit E Gasque P Nouvelot A MacKenzie ET Fontaine M Expression of receptors for complement anaphylatoxins C3a and C5a following permanent focal cerebral ischemia in the mouse Exp Neurol 2000 161 373 382 10683302 10.1006/exnr.1999.7273

[B52] Vedeler CA Matre R Peripheral nerve CR1 express in situ cofactor activity for degradation of C3b J Neuroimmunol 1990 26 51 6 2403574 10.1016/0165-5728(90)90119-8

[B53] Kolev MV Ruseva MM Harris CL Morgan BP Donev RM Implication of complement system and its regulators in Alzheimer’s disease Curr Neuropharmacol 2009 7 1 8 19721814 10.2174/157015909787602805 PMC2724661

[B54] Veerhuis R Histological and direct evidence for the role of complement in the neuroinflammation of AD Curr Alzheimer Res 2011 8 34 58 21143154 10.2174/156720511794604589

[B55] Johnson S Lampert-Etchells M Pasinetti GM Rozovsky I Finch CE Complement mRNA in the mammalian brain: responses to Alzheimer’s disease and experimental brain lesioning Neurobiol Aging 1992 13 641 648 1362796 10.1016/0197-4580(92)90086-d

[B56] Singhrao SK Neal JW Morgan BP Gasque P Increased complement biosynthesis by microglia and complement activation on neurons in Huntigton’s diseaseexp neurol 1999 159 362 376 10506508 10.1006/exnr.1999.7170

[B57] Rogers J O’Barr S Tanzi R, Wasco W, Totowa NJ Inflammatory mediators in Alzheimer’s disease Molecular Mechanisms of Dementia 1997 Totowa, NJ: Human Press Inc 177 198

[B58] Loeffler DA Camp DM Bennett DA Plaque complement activation and cognitive loss in Alzheimer’s disease J Neuroinflammation 2008 5 9 18334032 10.1186/1742-2094-5-9 PMC2292690

[B59] Benoit ME Hernandez MX Dinh ML Benavente F Vasquez O Tenner AJ C1q-induced LRP1B and GPR6 proteins expressed early in Alzheimer disease mouse models, are essential for the C1q-mediated protection against amyloid-β neurotoxicity J Biol Chem 2013 288 654 65 23150673 10.1074/jbc.M112.400168 PMC3537064

[B60] Webster S Glabe C Rogers J Multivalent binding of complement protein C1q to the amyloid beta-peptide (A beta) promotes the nucleation phase of A beta aggregation Biochem Biophys Res Commun 1995 217 869 875 8554610 10.1006/bbrc.1995.2852

[B61] Boyett KW DiCarlo G Jantzen PT Jackson J O’Leary C Wilcock D Morgan D Gordon MN Increased fibrillar beta-amyloid in response to human clq injections into hippocampus and cortex of APP+PS1 transgenic mice Neurochem Res 2003 28 83 93 12587666 10.1023/a:1021600212829

[B62] Hoarau JJ Krejbich-Trotot P Jaffar-Bandjee MC Das T Thon-Hon GV Kumar S Neal JW Gasque P Activation and control of CNS innate immune responses in health and diseases: a balancing act finely tuned by neuroimmune regulators (NIReg) CNS Neurol Disord: Drug Targets 2011 10 25 43 21143144 10.2174/187152711794488601

[B63] Davies A Simmons DL Hale G Harrison RA Tighe H Lachmann PJ Waldmann H CD59, an LY-6-like protein expressed in human lymphoid cells, regulates the action of the complement membrane attack complex on homologous cells J Exp Med 1989 170 637 654 2475570 10.1084/jem.170.3.637 PMC2189447

[B64] Meri S Morgan BP Davies A Danials RH Olavesen MG Waldmann H Lachmann PJ Human protectin (CD59), an 18,000-20,000 MW complement lysis restricting factor, inhibits C5b-8 catalysed insertion of C9 into lipid bilayers Immunology 1990 72 1 9 1698710 PMC1384213

[B65] Meri S Waldmann H Lachmann PJ Distribution of protectin (CD59), a complement membrane attack inhibitor, in normal human tissues Lab Invest 1991 65 532 537 1721667

[B66] Powell MB Marchbank KJ Rushmere NK van den Berg CW Morgan BP Molecular cloning, chromosomal localization, expression, and functional characterization of the mouse analogue of human CD59 J Immunol 1997 1997 158 1692 702 9029105

[B67] Kooyman DL Byrne GW McClellan S Nielsen D Tone M Waldmann H Coffman TM McCurry KR Platt JL Logan JS In vivo transfer of GPI-linked complement restriction factors from erythrocytes to the endothelium Science 1995 269 89 92 7541557 10.1126/science.7541557

[B68] Gasque P Ischenko A Legoedec J Mauger C Schouft MT Fontaine M Expression of the complement classical pathway by human glioma in culture J Biol Chem 1993 268 25068 25074 8227070

[B69] Morgan D Diamond DM Gottschali PE Ugen KE Dickey C Hardy J Duff K Jantzen P DiCarlo G Wilcock D Connor K Hatcher J Hope C Gordon M Arendash GW A beta peptide vaccination prevents memory deficits in an animal model of Alzheimer disease Nature 2000 408 982 985 11140686 10.1038/35050116

[B70] Morgan BP Complement regulatory molecules: application to therapy and transplantation Immunol Today 1995 16 257 9 7544975 10.1016/0167-5699(95)80175-8

[B71] Klegeris A Schwab C Bissonnette CJ McGeer PL Induction of complement C9 messenger RNAs in human neuronal cells by inflammtory stimuli: relevance to neurodegenerative disorders Exp Gerontol 2001 36 1179 1188 11404058 10.1016/s0531-5565(00)00265-5

[B72] Crehan H Hardy J Pocock J Microglia, Alzheimer’s disease, and complement. Int J Alzheimers Dis 2012 2012 983640 doi: 10.1155/**2012**/983640. Epub **2012** Aug 21 22957298 10.1155/2012/983640 PMC3432348

[B73] Gál P Dobó J Beinrohr L Pál G Závodszky P Inhibition of the serine proteases of the complement system Adv Exp Med Biol 2013 735 23 40 23402017 10.1007/978-1-4614-4118-2_2

[B74] Schapira M Patston PA Serine protease inhibitors (serpins) Trends Cardiovasc Med 1991 1 146 51 21239316 10.1016/1050-1738(91)90019-B

[B75] Carrell RW Boswell DR Barrett A, Salvesen G In C1 Esterase Inhibitors (Human), Volume 12 1986 Amsterdam Elsevier: Chapter: Proteinase Inhibitors 403 420

[B76] Carter PE Dunbar B Fothergill JE Genomic and cDNA cloning of the human C1INH Eur J Biochem 1988 173 163 169 3267220 10.1111/j.1432-1033.1988.tb13980.x

[B77] de Zwaan C Kleine AH Diris JH Glatz JF Wellens HJ Strengers PF Tissing M Hack CE van Dieijen-Visser MP Hermens WT Continuous 48-h C1-inhibitor treatment, following reperfusion therapy, in patients with acute myocardial infarction Eur Heart J 2002 23 1670 7 12398824 10.1053/euhj.2002.3191

[B78] Vedeler CA Matre R Sadallah S Schifferli J Soluble complement receptor type 1 in serum and cerebrospinal fluid of patients with Guillain-Barré syndrome and multiple sclerosis J Neuroimmunol 1996 67 17 20 8707926 10.1016/0165-5728(96)00035-5

[B79] Weisman HF Bartow T Leppo MK Soluble human complement receptor type 1: in vivo inhibitor of complement suppressing post-ischemic myocardial inflammation and necrosis Science 1990 249 146 151 2371562 10.1126/science.2371562

[B80] Naj AC Jun G Beecham GW Wang LS Vardarajan BN Buros J Gallins PJ Buxbaum JD Jarvik GP Crane PK Larson EB Bird TD Boeve BF Graff-Radford NR De Jager PL Evans D Schneider JA Carrasquillo MM Ertekin-Taner N Younkin SG Cruchaga C Kauwe JS Nowotny P Kramer P Hardy J Huentelman MJ Myers AJ Barmada MM Demirci FY Baldwin CT Common variants at MS4A4/MS4A6E, CD2AP, CD33 and EPHA1 are associated with late-onset Alzheimer’s disease Nat Genet 2011 43 436 41 21460841 10.1038/ng.801 PMC3090745

[B81] Hazrati LN Van Cauwenberghe C Brooks PL Brouwers N Ghani M Sato C Cruts M Sleegers K St George-Hyslop P Van Broeckhoven C Rogaeva E Genetic association of CR1 with Alzheimer’s disease: a tentative disease mechanism Neurobiol Aging 2012 33 2949 e5-2949.e12 22819390 10.1016/j.neurobiolaging.2012.07.001

[B82] Almeda S Rosenberg RD Bing DH The binding properties of human complement component C1q: interaction with mucopolysaccharides J Biol Chem 1983 258 785 791 6218163

[B83] Matsuoka Y Picciano M Malester B LaFrancois J Zehr C Daeschner JM Olschowka JA Fonseca MI O’Banion MK Tenner AJ Lemere CA Duff K Inflammatory responses to amyloidosis in a transgenic mouse model of Alzheimer’s disease Am J Pathol 2001 158 1345 1354 11290552 10.1016/S0002-9440(10)64085-0 PMC1891893

[B84] Sárvári M Vágó I Wéber CS Nagy J Gál P Mák M Kósa JP Závodszky P Pázmány T Inhibition of C1q-beta-amyloid binding protects hippocampal cells against complement mediated toxicity J Neuroimmunol 2003 137 12 8 12667643 10.1016/s0165-5728(03)00040-7

[B85] Merline R Schaefer RM Schaefer L The matricellular functions of small leucine-rich proteoglycans (SLRPs) J Cell Commun Signal 2009 3 323 35 19809894 10.1007/s12079-009-0066-2 PMC2778586

[B86] Kirschfink M Blasé L Engelmann S Schwartz-Albiez R Secreted chondroitin sulfate proteoglycan of human B cell lines binds to the complement protein C1q and inhibits complex formation of C1 J Immunol 1997 158 1324 1331 9013976

[B87] Botto M Dell’Agnola C Bygrave AE Thompson EM Cook HT Petry F Loos M Pandolfi PP Walport MJ Homozygous C1q deficiency causes glomerulonephritis associated with multiple apoptotic bodies Nat Genet 1998 19 56 9 9590289 10.1038/ng0598-56

[B88] Fujita Y Inoue I Inagi R Miyata T Shinzato T Sugiyama S Miyama A Maeda K Inhibitory effect of FUT-175 on complement activation and its application for glomerulonephritis with hypocomplementemia Nihon Jinzo Gakkai Shi 1993 35 393 7 8341019

[B89] Kapil A Sharma S Immunopotentiating compounds from *Tinospora cordifolia* J Ethnopharmacol 1997 58 89 95 9406896 10.1016/s0378-8741(97)00086-x

[B90] Kapil A Moza N Anticomplementary activity of boswellic acids—an inhibitor of C3-convertase of the classical complement pathway Int J Immunopharmacol 1992 14 1139 1143 1452399 10.1016/0192-0561(92)90048-p

[B91] Holmquist E Okroj M Nodin B Jirström K Blom AM Sushi domain-containing protein 4 (SUSD4) inhibits complement by disrupting the formation of the classical C3 convertase FASEB J 2013 27 2355 66 23482636 10.1096/fj.12-222042

[B92] Duarte ME Cardoso MA Noseda MD Cerezo AS Structural studies on fucoidans from the brown seaweed Sargassum stenophyllum Carbohydr Res 2001 333 281 93 11454335 10.1016/s0008-6215(01)00149-5

[B93] Rocha HA Franco CR Trindade ES Caryalho LC Veiga SS Leite EL Dietrich CP Nader HB A fucan from the brown seaweed Spatoglossum schroederi inhibits Chinese hamster ovary cell adhesion to several extracellular matrix proteins Braz J Med Biol Res 2001 34 621 6 11323748 10.1590/s0100-879x2001000500009

[B94] Blondin C Fisher E Boisson-Vidal C Kazatchkine MD Jozefonvicz J Inhibition of complement activation by natural sulfated polysaccharides (fucans) from brown seaweed Mol Immunol 1994 31 247 253 7908118 10.1016/0161-5890(94)90121-x

[B95] Zabel M Schrag M Crofton A Tung S Beaufond P Van Ornam J Dininni A Vinters HV Coppola G Kirsch WM A Shift in microglial β-amyloid binding in Alzheimer’s disease is associated with cerebral amyloid angiopathy Brain Pathol 2013 23 390 401 23134465 10.1111/bpa.12005 PMC3586773

[B96] Gerard NP Gerard C The chemotactic receptor for human C5a anaphylatoxin Nature 1991 349 614 617 1847994 10.1038/349614a0

[B97] Mukherjee P Pasinetti GM Complement anaphylatoxin C5a neuroprotects through mitogen-activated protein kinase-dependent inhibition of caspase 3 J Neurochem 2001 77 43 9 11279260 10.1046/j.1471-4159.2001.00167.x

[B98] Osaka H Mukherjee P Aisen PS Pasinetti GM Complement-derived anaphylatoxin C5a protects against glutamate-mediated neurotoxicity J Cell Biochem 1999 73 303 11 10321830

[B99] Bard F Cannon C Barbour R Burke RL Games D Grajeda H Guido T Hu K Huang J Johnson-Wodd K Peripherally administered antibodies against amyloid beta peptide enter the central nervous system and reduce pathology in a mouse model of Alzheimer disease Nat Med 2001 6 916 919 10932230 10.1038/78682

[B100] Castro JL Broughton HB Russell MG Rathbone D Watt AP Ball RG Chapman KL Patel S Smith AJ Marshall GR Matassa VG 5-(Piperidin-2-yl)- and 5-(homopiperidin-2-yl)-1,4-benzodiazepines: high-affinity, basic ligands for the cholecystokinin-B receptor J Med Chem 1997 40 2491 2501 9258356 10.1021/jm9608523

[B101] Wong AK Finch AM Pierens GK Craik DJ Taylor SM Fairlie DP Small molecular probes for G-protein-coupled C5a receptors: conformationally constrained antagonists derive from the C terminus of the human plasma protein C5a J Med Chem 1998 41 3417 3425 9719594 10.1021/jm9800651

[B102] Zhang X Boyar W Galakatos N Gonnella NC Solution structure of a unique C5a semi-synthetic antagonist: implications in receptor binding Protein Sci 1997 6 65 72 9007977 10.1002/pro.5560060107 PMC2143522

[B103] Whiss PA Pexelizumab Alexion Curr Opin Investig Drugs 2002 3 870 877 12137406

[B104] Ager RR Fonseca MI Chu SH Sanderson SD Taylor SM Woodruff TM Tenner AJ Microglial C5aR (CD88) expression correlates with amyloid-beta deposition in murine models of Alzheimer’s disease J Neurochem 2010 113 389 401 20132482 10.1111/j.1471-4159.2010.06595.x PMC2921960

[B105] Chitnis T Imitola J Wang Y Elyaman W Chawla P Sharuk M Raddassi K Bronson RT Khoury SJ Elevated neuronal expression of CD200 protects Wlds mice from inflammation-mediated neurodegeneration Am J Pathol 2007 170 1695 712 17456775 10.2353/ajpath.2007.060677 PMC1854964

[B106] Xing C Lee S Kim WJ Jin G Yang YG Ji X Wang X Lo EH Role of oxidative stress and caspase 3 in CD47-mediated neuronal cell death J Neurochem 2009 108 430 6 19012741 10.1111/j.1471-4159.2008.05777.x PMC3705576

[B107] Wang Y Li Y Dalle Lucca SL Simovic M Tsokos GC Dalle Lucca JJ Decay accelerating factor (CD55) protects neuronal cells from chemical hypoxia-induced injury J Neuroinflammation 2010 7 24 20380727 10.1186/1742-2094-7-24 PMC2867804

[B108] Kolev MV Tediose T Sivasankar B Harris CL Thome J Morgan BP Donev RM Upregulating CD59: a new strategy for protection of neurons from complement-mediated degeneration Pharmacogenomics J 2010 10 12 9 19884909 10.1038/tpj.2009.52

[B109] Griffiths MR Neal JW Fontaine M Das T Gasque P Complement factor H, a marker of self protects against experimental autoimmune encephalomyelitis J Immunol 2009 182 4368 77 19299737 10.4049/jimmunol.0800205

[B110] Aiyaz M Lupton MK Proitsi P Powell JF Lovestone S Complement activation as a biomarker for Alzheimer’s disease Immunobiology 2012 217 204 15 21856034 10.1016/j.imbio.2011.07.023

[B111] Wang Y Hancock AM Bradner J Chung KA Quinn JF Peskind ER Galasko D Jankovic J Zabetian CP Kim HM Leverenz JB Montine TJ Ginghina C Edwards KL Snapinn KW Goldstein DS Shi M Zhang J Complement 3 and factor h in human cerebrospinal fluid in Parkinson’s disease, Alzheimer’s disease, and multiple-system atrophy Am J Pathol 2011 178 1509 16 21435440 10.1016/j.ajpath.2011.01.006 PMC3078443

[B112] Daborg J Holmgren S Abramsson A Andreasson U Zetterberg M Nilsson S Minthon L Skoog I Blennow K Pekna M Hanse E Zetterberg H Complement gene single nucleotide polymorphisms and biomarker endophenotypes of Alzheimer’s disease J Alzheimers Dis 2013 35 51 7 23313922 10.3233/JAD-121930

[B113] Hampel H Shen Y Beta-site amyloid precursor protein cleaving enzyme 1 (BACE1) as a biological candidate marker of Alzheimer’s disease Scand J Clin Lab Invest 2009 69 8 12 18609117 10.1080/00365510701864610

[B114] Singh S Kushwah AS Singh R Farswan M Kaur R Current therapeutic strategy in Alzheimer’s disease Eur Rev Med Pharmacol Sci 2012 16 1651 64 23161037

[B115] Janus C Pearson J McLaurin J Mathews PM Jiang Y Schmidt SD Chlshti MA Horne P Hestlin D French J A beta immunization reduces behavioral impairment and plaques in a mode of Alzheimer disease Nature 2000 408 979 982 11140685 10.1038/35050110

[B116] Schenk D Barbour R Dunn W Gordon G Grajeda H Guido T Hu K Huang J Johnson-Wood K Khan K Immunization with amyloid-beta attenuates Alzheimer disease-like pathology in the PDAPP mouse Nature 1999 40 173 177 10408445 10.1038/22124

[B117] Lambracht-Washington D Rosenberg RN Active DNA Aβ42 vaccination as immunotherapy for Alzheimer disease Transl Neurosci 2012 3 307 313 23741624 10.2478/s13380-012-0037-6 PMC3670794

[B118] Greenberg SM Bacskai BJ Hyman BT Alzheimer disease’s double-edged vaccine Nat Med 2003 9 389 97 12640449 10.1038/nm847

[B119] McGeer PL McGeer E Is there a future for vaccination as a treatment for Alzheimer’s disease? Neurobiol Aging 2003 24 391 5 12600715 10.1016/s0197-4580(02)00157-4

[B120] Nicoll JAR Wilkinson D Holmes C Steart P Markham H Weller RO Neuropathology of human Alzheimer disease after immunization with amyloid beta peptide: a case report Nat Med 2003 9 448 452 12640446 10.1038/nm840

[B121] Panza F Frisardi V Solfrizzi V Imbimbo BP Logroscino G Santamato A Greco A Seripa D Pilotto A Immunotherapy for Alzheimer’s disease: from anti-β-amyloid to tau-based immunization strategies Immunotherapy 2012 4 213 38 22339463 10.2217/imt.11.170

[B122] Vellas B Carrillo MC Sampaio C Brashear HR Siemers E Hampel H Schneider LS Weiner M Doody R Khachaturian Z Cedarbaum J Grundman M Broich K Giacobini E Dubois B Sperling R Wilcock GK Fox N Scheltens P Touchon J Hendrix S Andrieu S Aisen P EU/US/CTAD task force members: designing drug trials for Alzheimer’s disease: what we have learned from the release of the phase III antibody trials: a report from the EU/US/CTAD task force Alzheimers Dement 2013 9 438 44 23809364 10.1016/j.jalz.2013.03.007

[B123] Farlow M Arnold SE van Dyck CH Aisen PS Snider BJ Porsteinsson AP Friedrich S Dean RA Gonzales C Sethuraman G DeMattos RB Mohs R Paul SM Siemers ER Safety and biomarker effects of solanezumab in patients with Alzheimer’s disease Alzheimers Dement 2012 8 261 71 22672770 10.1016/j.jalz.2011.09.224

[B124] DeMottos RB Bales KR Cummins DL Dodart JC Paul SM Hotltzman DM Peripheral anti-A beta antibody alters CNS and plasma A beta clearance and decreases brain A beta burden in a mouse model of Alzheimer’s disease Proc Natl Acad Sci U S A 2001 98 8850 8855 11438712 10.1073/pnas.151261398 PMC37524

[B125] Gandy S Molecular basis for anti-amyloid therapy in the prevention and treatment of Alzheimer’s disease Neurobiol Aging 2002 23 1009 1016 12470796 10.1016/s0197-4580(02)00125-2

[B126] Weiner HL Selkoe DJ Inflammation and therapeutic vaccination in CNS diseases Nature 2002 420 879 84 12490962 10.1038/nature01325

[B127] Sigurdsson EM Wisniewski T Frangione B A safer vaccine for Alzheimer’s disease? Neurobiol Aging 2002 23 1001 8 12470795 10.1016/s0197-4580(02)00124-0

[B128] Monsonego A Nemirovsky A Harpaz I CD4 T cells in immunity and immunotherapy of Alzheimer’s disease Immunology 2013 139 438 46 23534386 10.1111/imm.12103 PMC3719061

